# The impact of widowhood on mental health: anxiety, depression, and stress among widowed women in Nepal

**DOI:** 10.3389/fgwh.2024.1256484

**Published:** 2024-07-23

**Authors:** Pooja Mamidanna, Narendra Singh Thagunna, Jyotshna Dangi, Diane C. Zelman

**Affiliations:** ^1^ Clinical Psychology PhD Program, Alliant International University San Francisco Bay Area, Emeryville, CA, United States; ^2^ Department of Psychology, K&K International College, Tribhuvan University, Kathmandu, Nepal; ^3^ Department of Psychology, Padma Kanya Multiple Campus, Tribhuvan University, Kathmandu, Nepal; ^4^ Department of Research, Psychdesk Foundation, Kathmandu, Nepal; ^5^ Department of Counselling, Sainik Awasiya Mahavidyalaya, Tribhuvan University, Bhaktapur, Nepal

**Keywords:** widowhood, widow, Nepal, South Asian mental health, anxiety, depression, stress, cross-sectional

## Abstract

**Introduction:**

In South Asia, particularly in regions with strong patriarchal norms, widowhood is stigmatized, compounding the negative impact of grief and partner loss. This study measured the prevalence of mental health symptoms among widows in Nepal and its relationship to demographic variables.

**Methods:**

This cross-sectional study surveyed 588 Nepalese widows from six districts in Nepal (mean age = 52.62, SD = 13.99) who had lost their spouses within the past two years. Participants completed the Anxiety, Depression and Stress Scale (ADSS). Analyses examined prevalence of anxiety, depression, and stress symptoms, using standard ADSS cut-points. Level of anxiety, depression, and stress symptoms measured by the ADSS in the sample were also compared with female psychiatric and nonpsychiatric normative ADSS data, and were compared with one available comparison sample (a sample of older Nepalese women). Measures of association between ADSS scores and demographic variables were computed.

**Results:**

Results showed that a high percentage of the Nepalese widows reported moderate to severe symptoms of anxiety, depression, and stress. They also endorsed significantly higher levels of anxiety, depression, and stress symptoms relative to normative data and the comparison sample. Stress scores were significantly negatively correlated with age, Anxiety and Depression scores were associated with income under the poverty line, and Depression scores were associated with homemaker status.

**Discussion:**

These findings confirm the high emotional distress among widowed women in Nepal, and establish the relationship between emotional distress and poverty, homemaker status, and age. These findings can inform public health efforts and mental health care providers regarding the mental health needs of widows in Nepal.

## Introduction

In Nepal, widows account for greater than 4.6 percent of the female population (1), with more than 218,040 widows in the 2011 Nepal national census. According to the 12th National Census in Nepal, the number of single women following marriage (which includes both widows and divorced/separated women) rose from 1.5 percent of the female population in 2011 to 7.3 percent in 2021 (2). The prevalence of young widowhood has increased over the past two decades due to natural disasters, illness, poverty, and political conflicts such as the decade-long People's War (1996–2006) (2, 4). Periods of war and civil unrest disproportionately affect the physical and mental health of vulnerable populations such as women, children, refugees, widows, orphans, and the elderly (5, 6). Research focusing on widows in Nepal is essential to contribute to a broader knowledge of how such social circumstances affect the mental health of marginalized populations, thereby potentially advancing mental health equity in conflict-affected regions.

Multiple conditions contribute to the low social status, stigma, and vulnerability of widows in Nepal. Most Nepalese women married young, are homemakers, are responsible for all child rearing, and were totally financially dependent on their husbands (4, 7, 8). In addition, 86% of Nepalese widows have minimal formal education (7).

Widows in Nepal face disenfranchisement and stigma based on religious and cultural traditions and influenced by traditional female gender roles (9). In traditional South Asian cultures, women encounter a patriarchal system in which a woman's identity is defined by her obligations to her family and husband; women do not have an independent identity apart from their own families, husbands, in-laws, and extended families (9). The word for widow, विधवा (Vidhavā), which is derived from a Sanskrit word that means “empty,” is perceived as pejorative (10). In South Asian culture, widowhood is believed to make women unlucky, and widows may be directly or indirectly blamed for the death of their spouses. Since Hindu cultural and religious practices consider widows inauspicious, they are expected to conform to specific norms including in their behaviors and attire. Hindu practice forbids widows from wearing the color red, a symbol of good fortune in religious ritual; instead, they are expected to wear only the color white (10). They are banned from attendance in temples and at certain religious and cultural ceremonies, such as weddings and child hair cutting ceremonies. Finally, they are excluded from certain worship of gods and goddesses, and they may not remarry. According to the sati system, a historical Hindu practice over a hundred years ago, widows were burned alive simultaneously on the pyre with their husbands' bodies during their husband's funerals (11). Although sati was banned in Nepal in the 1920s (12), widows continue to experience lasting forms of marginalization.

The foremost issues confronting widows in Nepal include economic adversity, mental distress caused by family and society, lack of independence, and denial of socioeconomic rights (13). Uprety and Adhikary (10) noted that “social norms restrict their mobility, remarriage, employment, interest, happiness, ownership, and other social and cultural relationships”. Young widows cannot depend on government resources when dealing with unforeseen financial hardships or their change in social status (4, 14–16). There is also a documented lack of public policy initiatives with regard to housing, access to medical care, and mental health services (4).

Tiwari and Bhattarai (13) reported that the increasing number of widows has resulted in poverty and illiteracy. Since most Nepalese widows have little education, married young, and take on the role of a homemaker, they do not have the opportunity to pursue higher education; thus, when their husbands die, they are faced with severe economic distress (10). Meanwhile, they need to utilize whatever resources they have to pay for their children's education. Additionally, many widows do not have the paperwork necessary to claim their deceased husband's property, or they face barriers obtaining financial grants due to their marriages being unregistered, which is typical in Nepal (4, 15). Despite the existence of community resources to support widows (e.g., Women for Human Rights), current circumstances leave widows vulnerable and socially isolated (4, 7, 15, 16).

Sabri et al. (15) highlight the heightened vulnerability of widows in Nepal, who are often blamed for their husbands' deaths. This vulnerability is further exacerbated by their disenfranchisement and lack of resources (13). Numerous studies have highlighted the high risk of violence faced by widows in Nepal (9, 17, 18), with one study estimating that 78% have experienced violence (1). A study of 27 Nepalese widows yielded accounts of violent encounters, including psychological, physical, and sexual assault from both family and community members (15). The authors reported that women managed abusive experiences adaptively, such as by utilizing social support and addressing verbal conflict directly, and maladaptively, such as through suicidal ideation or substance use (15).

An interview and focus group study reported that to shield themselves from stigma and discrimination and to protect their children, some widows in Nepal do not disclose their widow status to the community (16). Respondents also indicated they feared sexual harassment or assault from members of the community and even health care providers. They also described remaining silent due to their unresolved and prolonged bereavement and because they felt their children were too young to understand grief. Similar outcomes were reported in an interview study of 42 Nepali widows from the Kathmandu Valley and Surketh districts, who reported a lack of emotional and instrumental support from their late husband's family and community, creating susceptibility to abuse and economic uncertainty for themselves and their children (14).

These studies underscore the experiences of stigma and discrimination, risk of abuse, challenges in coping with grief, and financial concerns experienced by Nepalese widows. Several studies have shown that for these reasons, Nepalese widows are at high risk of developing mental health problems (9, 17, 18). A study of 358 Nepali war widows reported that 53% of the widows presented with depression symptoms and 63% presented with anxiety symptoms on the Beck Anxiety and Depression Inventories (17). A more recent study evaluated symptoms of posttraumatic stress, social support, and suicidal ideation among 204 Nepali widows whose husbands died in the year prior, using the Hopkins Symptoms Checklist-25, PTSD Checklist-Civilian Version, Somatic Symptom Scale-8, and the Multidimensional Scale of Perceived Social Support, reporting that 30.4% of the widows experienced suicidal ideation during their lifetime and 16.2% reported suicidal ideation in the past year (19). Widows who experienced suicidal ideation in the last year also scored significantly higher on measures of anxiety, depression, PTSD, somatic symptoms, and prolonged grief, as well as neurological complaints. Finally, a sociocultural exploration of the symptoms of Persistent Complex Bereavement Disorder (PCBD) among Nepalese widows concluded that the construct was similar to DSM-5 PCBD, although there was no one phrase used for bereavement across the sample (20). Factors associated with complicated bereavement included having experienced torture during war-related combat, economic and familial pressures, and experience of prejudice. The prevalence of suicidal ideation was a surprising 31% and 62% in the preceding year and lifetime, respectively (20).

To summarize, the suffering experienced by widows in Nepal is multilayered, including stigmatization based on female gender in a patriarchal society, discrimination based on religious and cultural practice, socioeconomic distress, and risk of sexual and physical victimization (4, 7, 9, 13–16). Nepal represents a multitude of religious, caste and socioeconomic groups across 77 districts of Nepal, but current research on widowhood has been restricted to limited population surveys in one or two districts of Nepal and small-sample interview studies. This body of research, much of which uses qualitative methodology and has emerged from the laboratory of Pamela J. Surkan at Johns Hopkins University [e.g., (15, 18, 19)] and others (9, 17) has shown that widowhood is associated with anxiety, depression, stress, posttraumatic symptoms, and suicidality. Furthermore, existing studies show that widows in Nepal have insufficient access to appropriate mental health and physical health care (7, 14–16). There is a need for research on in a variety of districts and exploration of the impact of demographic characteristics (e.g., gender, age, socio-economic status, and caste) on mental health symptoms among widows in Nepal (15, 17).

The current study addresses a number of gaps in current knowledge on the mental health impact of widowhood in Nepal. As discussed above, existing research on widowhood in Nepal has used only small sample studies or sampling of only one or two urban districts in Nepal, and there is a need to represent the experiences of a larger part of the population and both urban and rural provinces. Second, although existing research in these limited samples has reported anxiety, depression, and stress, the studies do not use comparison samples or reference to South Asian normative data, showing the need to evaluate the extent of mental health concerns relative to established benchmarks or normative samples and comparison samples. Finally, there is a need to evaluate the relationship between mental health concerns and demographic variables, because such characteristics as age, socioeconomic status, and caste can affect access to resources, social support networks, and coping strategies. To address these gaps in the literature this study assesses the degree of anxiety, depression, and stress symptoms among a large sample of widows in Nepal across a wide variety of districts in rural and urban areas, and its relationship with demographic and cultural variables. It utilizes the Anxiety, Depression and Stress Scale [ADSS; (21)], a scale validated within South Asia. Levels of anxiety, depression, and stress among the current sample of widows are interpreted (1) relative to the ADSS scale cut-points, which can be used to indicate percentages of individuals in a sample who experience mild, moderate, and severe symptoms, (2) by comparison with mean anxiety, depression and stress scores in female general population normative data provided in the ADSS manual, (3) by comparison with mean anxiety, depression and stress scores in female psychiatric population normative data provided in the ADSS manual, and (4) by comparison with mean ADSS scores for older female women in Nepal. Therefore, this is the first study to evaluate level of mental health concerns among widows in Nepal who are from a variety of urban and rural provinces, using cut-points to estimate levels of severity of mental health concerns, and considering whether mental health concerns are greater than those in normative data and an existing Nepalese data sample.

## Methods

### Participants

Participants were 588 adult women widowed within the past two years, from six districts of five provinces in Nepal (Kailali district from Sudurpashim Province, Surketh from Karnali Province Dang and Kapilvastu districts from Lumbani province, Kavereplanchok district from Bagmati province, and Bara district from Madhesh Province). These districts were sampled because they represent a range of urban and rural communities, as well as a variety of socioeconomic and educational levels and sub-castes, and because research assistants who spoke the local dialect were available to administer study measures. Participants from the following communities: Magar, Tharu, Gurung, Newar, Tamang, Rai, and Limbu belong to indigenous communities; these participants received the label “tribal/indigenous” for further analyses. Participants self-describing as Brahmin, Chhetri and Dalit belong to the caste system; they were labelled as “non-tribal” for further analyses. The study used a non-probability convenience sampling strategy including snowball sampling. Inclusion criteria were majority status (at least 18 years of age) and being widowed within the past two years, and potential participants were excluded if a participant indicated they had a severe mental health condition or reported being on medication for mental health problems.

Participants provided oral consent to the research assistant at the time of participation. The second author (Dr. Thagunna) received permission to recruit participants from the research department of K & K International College (Nepal), and the present research protocol was approved by the Institutional Review Board (IRB) of Alliant International University.

## Measures

### Anxiety, depression and stress scale (ADSS-BSPSA)

Since anxiety, depression and stress symptoms were the outcomes of interest, the study utilized the Anxiety, Depression and Stress Scale (ADSS-BSPSA). It was developed and validated in India (21). The scale comprises 48 items divided into 3 scales: an Anxiety scale consisting of 19 items, a Depression scale consisting of 15 items and a Stress scale consisting of 14 items. Each item is scored 1 for “Yes” and is scored 0 for “No” responses, yielding scores of 0–19, 0–14 and 0–14 for the Anxiety, Depression and Stress Scales. An example of an item in the Anxiety scale is, “I feel more nervous and anxiety than usual.” An example of an item in the Depression scale is, “I feel sad and depressed.” An example of an item in the Stress scale is, “Stressful events cause problems in my relationships with other people.” The ADSS manual provides derived cut-points for mild, moderate, and severe levels for the Anxiety, Depression and Stress Scales, and these were used to characterize the current study sample. The authors of the ADSS also provide normative data for males and females for a non-psychiatric sample (*n = *972) and a psychiatric sample (*n = *205). Scores for the psychiatric standardization sample were gathered from males and females at three psychiatric hospitals in Lucknow, India. Scores for the non-psychiatric standardization sample were gathered from males and females from urban and rural settings of varied socioeconomic status, including rural sample of from Deva village in Uttar Pradesh, India, residents of a slum in Lucknow, Uttar Pradesh, India, and individuals from the village Kagzipur in Uttar Pradesh, and university postgraduate and undergraduate students from Lucknow University. The present study utilized the ADSS manual means and standard deviations for the Anxiety, Depression, and Stress Scales for the female non-psychiatric and psychiatric normative data. The ADSS manual provides evidence of construct validity of the ADSS via a principal factoring with VARIMAX factor analytic study conducted separately for the non-psychiatric and psychiatric samples, both yielding 3 factors in the Anxiety scale (physical symptoms, apprehension, dryness of mouth), 2 factors in the Depression scale (inertia-loss of interest and worth and poor emotional control), and 2 factors in the Stress scale (emotional arousal and negative life events) (22). Internal consistency reliability as measured by Cronbach's alpha was .76, .75 and .61 for the Anxiety, Depression, and Stress Scales, respectively (21). In the current study, Cronbach's alpha was .76, .77 and .72 for these three scales.

### Demographic characteristics

Demographic information collected from the women included age (in years), religious faith (Hindu, Christian, Buddhist, or other), months since widowhood, employment (agricultural, homemaker, business owner, other), education level (no formal education, and primary, secondary, and undergraduate education), caste (tribal/indigenous vs. non-tribal), and monthly income.

## Procedure

Data were gathered by undergraduate psychology research assistants at K & K International College who came from the districts of participants included in the study and who spoke the local dialect. The research assistant requested access to names and contact information of widows in the community from regional government municipality offices. Research assistants also contacted local community widow support groups for permission to recruit, and potential participants were also recruited by referrals from participants using snowball sampling. Research assistants contacted potential participants, determined interest and eligibility for participation, obtained oral informed consent, and arranged individual meetings with interested participants. At this meeting, a detailed description of the research purpose and rights of participants was provided. Research assistants administered the survey face-to-face or by phone in either the Nepali language or in the local district dialect. The languages/dialects used by research assistants to administer questionnaires were as follows: Kailali district (Tharu, Doteli, or Nepali), Dang and Kapilvastu districts (Awadhi, Tharu or Nepali), Kavrepalanchok district (Nepali), Bara (Bhojpuri, Maithill, or Nepali), and Surkhet district (Nepali or Magar). Participation took less than 15 min. For the participants who indicated they were illiterate, the research assistant verbally administered the questionnaire in the common dialect of the research assistant and the participant. For participants who indicated they were literate, the participant completed the questionnaire with pencil and paper.

## Data analysis

Data were analyzed with Statistical Package for Social Science (SPSS) version 21. Descriptive data for the ADSS was computed, including mean, SD*,* and Cronbach's alpha to confirm internal consistency of the ADSS scales. Normality was evaluated using the Kolmogorov-Smirnov test. Frequency and percentages for severity categories of stress, anxiety, and depression symptoms (none, mild, moderate, severe) in the widow sample were calculated based on ADSS cut-points provided in the manual (22). For the 19-item Anxiety scale, cut-points for the 19-item scale were 3, 5, and 10 for mild, moderate, and severe anxiety symptoms. For the 15-item Depression scale, cut-points were 2, 4, and 10 for mild, moderate, and severe depression symptoms. For the 14-item Stress scale, cut-points were 4, 6, and 10 for mild, moderate, and severe stress symptoms.

Anxiety, Depression and Stress scores of the widow sample were compared with two normative reference values and one comparison sample. First, scores for the widow sample were compared with ADSS manual normative data (means and standard deviations) for adult females in the general population (non-psychiatric) and for females receiving psychiatric care (22). One-sample *t-*tests were used to compare the means in the widow sample to the ADSS normative data for adult non-psychiatric and psychiatric females. The third comparison was with a sample of ADSS scores from Nepal obtained from other researchers for women between the ages of 60 and 98 years (*M = *69.24, SD* *=* *7.53*)*. Independent-sample *t-*tests were used to compare means of the widow sample with the Nepalese older women sample.

To explore relationships between ADSS scores and demographic variables, Pearson correlations were computed between ADSS scale scores and age and time since loss of spouse. ADSS scale scores were compared over groups based on selected dichotomous demographic variables (tribal status: tribal/indigenous vs. nontribal status, and social class: above and below poverty level), using the Mann-Whitney *U*-test. The Kruskal–Wallis *H*-test was used to compare ADSS scale scores over four occupational levels (agriculture, housewife, business, and other). Analyses of religion-based differences were not conducted due to the sample being primarily Hindu.

## Results

### Sample characteristics

Table 1 provides the sample characteristics. Participants reported they were widowed a mean of 4.48 months earlier (SD* *=* *4.71). Age of the sample ranged from 18 to 93 years (*M = *52.62, SD* *=* *13.99). Of the sample, 44% (*n *= 259) had no formal education, 43.7% (*n *= 257) had primary education (fifth through tenth grade), 9.7% (*n *= 57) had a secondary education (grade eleven and twelve), and 2.6% (*n *= 15) had an undergraduate degree. Mean monthly income ranged from 2,000 to 50,000 Nepalese rupee (*M = *8,327.12*,* SD* = *6,171.82). In Nepal, 2,000 rupee is the equivalent of $16.81 U.S. per month and 50,000 Nepalese rupee is the equivalent of $420.19 U.S. per month. To place this into perspective, according to the Nepal Development Update, an estimated 31.2 percent of the Nepali population earn wages near the poverty line the poverty line (between $1.90 and $3.20 U.S. per day) and are at high risk of falling into extreme poverty (23).

**Table 1:** Demographic characteristics of Nepalese widowed women (*N *= 588).

Characteristic	*N*	%
Socioeconomic status (income)^a^		
Above the poverty line	322	56.46
Below the poverty line	256	43.54
Education		
No formal education	259	44.00
Primary education	257	43.70
Secondary education	57	9.70
Undergraduate degree	15	2.60
Religious Faith		
Hindu	410	83.67
Muslim	52	10.61
Buddhist	19	3.87
Christian	9	2.19
Indigenous/Tribal Status		
Tribal-Indigenous/Non-Tribal	219/369	37.20/62.80
Occupation		
In home	203	34.50
Out of home	385	65.50
Occupation		
Agriculture	290	49.30
Housewife	203	34.50
Business	51	8.70
Other	44	7.50
District & Province		
Bara Madhesh	100	17.00
Dang Lumbani	100	17.00
Kailali Sudurpashim	101	17.20
Kapilvastu Lumbini	100	17.00
Kavre Bagmati	100	17.00
Surketh Karnili	87	14.80

^a^ Poverty line based on Huaxia (23).

Regarding religious faith, 83.67% (*n = *410) of the sample were Hindu, 10.61% of the sample (*n = *52) were Muslim, 3.87% (*n = *19) were Buddhist, and 2.19% (*n = *9) of the sample were Christian. Participants reported that they are of the following tribal/indigenous and non-tribal sub-castes: 62.37% non-tribal (*n = *368), and tribal/indigenous 37.46% (*n = *221). Reported occupations of the sample were as follows: 49.30% Agriculture (*n = *290), 34.50% Homemaker (*n = *203), 8.70% Business Owners (*n = *51), and Other 7.50% (*n = *44).

### ADSS scores in widows relative to ADSS manual cut-points

Table 2 presents descriptive data for the ADSS in the widow sample, and the frequencies and percentages of normal, medium, high, and severe symptoms scores for the Anxiety, Depression and Stress Scales for the study sample, based on the ADSS manual cut-points (21), also presented earlier here in the Methods section). These findings indicate that roughly 85%, 84%, and 77% of participants reported moderate to severe levels of anxiety, depression, and stress symptoms, respectively.

**Table 2:** Frequency levels of anxiety, depression, and stress symptoms in widows in Nepal (*N* = 588).^a^

ADSS Severity^b^	Anxiety	Depression	Stress
	% (*n*)	% (*n*)	% (*n*)
Normal	4.8 (28)	6.0 (35)	9.9 (58)
Mild	9.4 (55)	9.9 (58)	12.9 (76)
Moderate	26.4 (350)	41.7 (245)	32.0 (188)
Severe	59.5 (350)	3.65 (7.5)	45.2 (266)
ADSS mean (SD)	9.9 (4.09)	7.5 (3.65)	7.83 (3.07)

^a^ Based on ADSS manual cut-points (21).

^b^ ADSS, Anxiety, Depression and Stress Scale (21).

### ADSS scores in widows relative to reference data

#### Comparison with ADSS female non-psychiatric normative data

A one sample *t*-test indicated that Anxiety scores were significantly higher among the widow sample (*M = *9.49, SD* *=* *4.09) relative to ADSS female non-psychiatric normative data (*M = *7.18, SD* *=* *6.18), *t* (587) = 13.71, *p *< . 001, with a medium effect size (Cohen's *d* = 0.57). Depression scores were also significantly higher in the widow sample (*M *=* *7.51, SD* *=* *3.65) relative to the ADSS female non-psychiatric sample normative data (*M *=* *5.98, SD* *=* *5.98), *t* (587) = 10.15, *p *< .001, with a medium effect size (Cohen's *d* = 0.42). Finally, Stress scores were also significantly higher in the widow sample (*M *=* *7.83, SD* *=* *3.07), relative to the ADSS female non-psychiatric normative data (*M *= 7.05, SD* *=* *4.05), *t* (587) = 6.17, *p *< .001, with a small effect size (Cohen's *d *= 0.25). Therefore the widow sample scored higher relative to ADSS non-psychiatric normative data on measures of anxiety, depression, and stress.

#### Comparison with ADSS female psychiatric normative data

A one-sample *t-*test showed Anxiety scores were significantly higher among the widowed women (*M *= 9.49, SD* *= 4.09), relative to the ADSS female psychiatric normative data (*M *= 8.41, SD* *= 3.87), *t* (587) = 6.41, *p *< .001, with a small effect size (Cohen's *d* = 0.26). Depression scores were not significantly different between the widow sample (*M *= 7.51, SD =* *3.65) and the ADSS psychiatric normative data (*M *= 7.71*,* SD* *= 4.32), *t* (587) = 1.34, *p* = 0.181. However, Stress scores were significantly higher in the widow sample (*M *= 7.83*,* SD* *= 3.07) relative to the ADSS psychiatric normative data (*M *= 7.33*,* SD* *= 2.89), *t* (587) = 3.96, *p *< .001, with a small effect size (Cohen's *d *= 0.16). Therefore, the widow sample scored higher relative to ADSS psychiatric normative data on measures of anxiety and stress, but not depression.

#### Comparison with Nepalese older women data

An independent samples *t*-test showed significantly higher Anxiety scores in the widow sample (*M *= 9.49*,* SD* *= 4.09), relative to the Nepalese older women sample (*M *= 8.11*,* SD* *= 4.78), *t* (687) = 3.06, *p *= 0.002 with a small effect size (Cohen's *d* = 0.33). Depression scores were also significantly higher in the widow sample (*M *= 7.51, SD* *= 3.65) relative to the Nepalese older women sample (*M *= 5.40, SD* *= 4.22), *t* (687) = 5.24, *p* < 0.001 with a medium effect size (Cohen's *d *= 0.57). Finally, Stress scores were also significantly higher in the widow sample (*M *= 7.83, SD* *= 3.07) relative to the Nepalese older women sample (*M *= 4.09, SD* *= 3.62), *t* (687) = 11.02, *p *< .001, with a strong effect size (Cohen's *d* = 1.17).

These results show that the Nepalese widow sample reported higher anxiety, depression, and stress symptoms relative to both the ADSS normative female non-psychiatric sample and the Nepalese older women sample, and higher anxiety and stress symptoms relative to ADSS female psychiatric normative data.

### Relationship between mental health symptoms and demographic measures

#### ADSS scores, age, and recency of widowhood

Pearson's correlation indicated nonsignificant correlations between age and Anxiety [*r* (586)* *= .016, *p *= .697, *ns*], and age and Depression [*r* (586) = −.009, *p* = .826, *ns*]. However, there was a significant negative correlation between age and Stress [*r* (586) = −.132, *p *= .001], with a small effect size (*r^2 ^*= .017). Therefore, data show that older widows reported lower stress symptoms.

Pearson's correlation analyses indicated that there were no significant relationships between recency of widowhood and Anxiety [*r* (586) = .007, *p *= .684], Depression [*r* (586)* *= .058, *p *= .164], or Stress [*r* (586*)* = .054, *p *= .194].

#### ADSS scores and tribal/indigenous status

Anxiety, Depression, and Stress scores were compared between individuals from tribal/indigenous and non-tribal ethnicity in Nepal, using the conservative non-parametric Mann-Whitney *U* test. No significant differences between subjects in Anxiety, Depression, or Stress scores were found based on tribal/indigenous vs. non-tribal status (Anxiety: *Mdn *= 10.00 (tribal) vs. 10.00 (non-tribal), *U* = 39,462, *p* = .635; Depression: *Mdn* = 7.00 (tribal) vs. 8.00 non-tribal, *U* = 38,142, *p* = .254; Stress: *Mdn *= 8.00 (tribal) vs. 8.00 (non-tribal), *U* = 37,488, *p *= .141). These results indicate that tribal/indigenous status was not related to the levels of anxiety, depression, and stress symptoms.

#### ADSS scores and socioeconomic status

Anxiety, Depression, and Stress scores were compared between those participants with incomes above and below the poverty line using the non-parametric Mann-Whitney *U* test. Anxiety scores were significantly higher among those below the poverty line (*Mdn *= 10.00) relative to those above the poverty line (*Mdn *= 9.00)., *U* = 36,219, *p* = .002. Depression scores were also significantly higher among those below the poverty line (*Mdn *= 8.00), relative to those above (*Mdn *= 7.00), *U* = 34,741, *p* < .001. There was no significant difference in Stress scores between the subjects who were above *(Mdn *= 8.00) and below (*Mdn *= 8.00) the poverty line, *U* = 38,606, *p* = .056. These findings show that anxiety and depression symptoms among widows are higher among those living below the poverty line.

#### ADSS scores and occupation

The non-parametric Kruskal–Wallis test was used to compare Anxiety, Depression, and Stress scores across four levels of occupation (Agricultural, Homemaker, Business and Other). Results indicated a significant difference in Depression scores, KW = 9.50, *p *= .02. Women who indicated that they were homemakers endorsed significantly higher Depression scores (*M *= 8.15, SD* *= 3.20) than those indicating they worked in agriculture (*M *= 7.15, SD* *= 3.82), business (*M *= 7.41, SD* *= 3.93) and other (*M *= 7.02, SD* *= 3.87). No significant difference in Anxiety scores, KW = 6.00, *p *= .11, or Stress scores, KW = 2.34, *p *= .50 were found.

## Discussion

After an international effort launched by the Loomba Foundation, the United Nations General Assembly passed Resolution A/RES/65/189 in 2010 declaring an annual International Day of Widows (24). The goals of this resolution were to highlight the discrimination, economic distress and stigma faced by widows worldwide, and to promote widows' human rights and basic physical and mental health (25). Annual United Nations updates on the status of widows feature the needs of widows in certain geographical regions as well as after geopolitical events such as wars and forced migration or health crisis such as COVID-19. Among other recommendations, these updates emphasize the need for better collection of demographic and other data on widows in specific countries to promote the global recognition and support of widows (26). Recent research has suggested that deprivations associated with widowhood, rather than being universal, may be most pronounced in certain countries (e.g., China, Ghana, Indian, Russia, and South Africa, (27). South Asia appears to be an area of particular concern (28, 29). As most research in this area has used qualitative methodology, the goal of the present research was to gather quantitative data to document the mental health impacts of widowhood in Nepal across a diverse set of provinces and districts, and to assess the relationship between mental health impact and demographic variables.

This study assessed the degree of anxiety, depression, and stress among 588 widowed women from 6 districts of 5 provinces in Nepal. The findings emphasize the very high level of anxiety, depression, and stress symptoms among widows in Nepal relative to normative data and a local comparison sample, including female psychiatric normative data. These findings support existing research showing elevated depression and anxiety symptoms among widows (17, 19), and are particularly noteworthy given that higher symptoms are associated with an increased risk for PTSD, suicidality, and risk for victimization such as violence and human trafficking (9, 13, 17, 18)).

Additionally, the study analyzed the relationship between the degree of these mental health symptoms and demographic and cultural variables. As mental health symptoms were high across the sample, the limited range of scores challenged the detection of association between symptoms and demographic variables. However, it was found that Stress scores were significantly negatively correlated with age, Anxiety and Depression scores were associated with income under the poverty line, and Depression scores were associated with homemaker status. These findings are consistent with prior assertions that financial stress creates high burden and increased risk for depression and anxiety among Nepalese widows (10, 17), and that poverty and lack of formal education increases risk of depression and anxiety (13).

In terms of occupational status, widows who were homemakers had higher levels of depression symptoms relative to those whose occupation was agriculture, business owner and other occupation, suggesting that wage earning outside of the home may increase both emotional and financial status and reduce disenfranchisement. This finding contrasts with prior research reporting no significant effect of occupation on levels of anxiety and depression symptoms (17, 19). In addition, in this study, no relationship was found between the duration of widowhood and mental health symptoms, although the current study sample had been widowed only for an average of five and a half months. In other research reporting that length of widowhood was positively associated with anxiety, it is noteworthy that the sample had been widowed on average for ten years; evaluation of widows over a longer period of time will better assess the long-term impact of widowhood (19).

The use of local research assistants to deliver questionnaires in regional dialects is a clear strength of this study. However, interpretation of results is limited by the absence of an age-equivalent comparison group of Nepalese non-widows, as comparison data used in this research included Nepalese older women and ADSS normative data. However, the uniformly higher levels of emotional distress shown in the widow sample relative to all three reference groups, including a psychiatric women sample, verifies the high risk among widows, and the demographic analyses show that this risk exists across both tribal/indigenous and nontribal castes.

This study along with past research, establishes that anxiety, depression, and stress are significant among Nepalese widows, and has also established a number of related variables including age, poverty, and vocational and cultural disenfranchisement. It would be valuable to understand more about the route by which demographic variables might influence mental health symptoms. In particular, it is crucial to understand more about the interaction between social class and widowhood, since it is likely that widows of lower social class face greater economic need, fewer resources with which to thrive without a spouse, and greater risk of victimization. Future research might also directly measure experienced stigma to understand the degree to which stigma might mediate negative mental health outcomes among widows in Nepal. A study of the coping skills widows employs to cope with and heal from their bereavement may inform future interventions. Sabri et al. (15) suggested that psychosocial interventions should include increasing awareness of widows' unique presenting concerns and acknowledging cultural attitudes that negative impact widows' lives. Additionally, initiatives should be concentrated on emboldening widows' resiliency and developing good coping mechanisms (15).

### Clinical implications

At the individual level, these findings have a number of clinical implications. Physical and mental health practitioners who work with South Asian women for mental health symptoms should consider widowhood among the identities that influence presenting concerns. Other important characteristics to evaluate include social class and its impact. Practitioners should be familiar with case management, vocational services, and family care providers to address other needs that contribute to emotional concerns. Finally, when clinicians are considering the impact of recent grieving process on presenting symptoms, they need to be cognizant of how the length of widowhood may or may not predict emotional concerns—as both recent and lasting widowhood may be associated with negative emotional and societal impacts.

### Public policy implications to support widows in Nepal

It is imperative to consider policies, programs and support needed by Nepalese widows. A key nonprofit advocacy body for widow's rights in Nepal is the Women for Human Rights (WHR), founded by Lily Thapa in 1994 (7, 15, 16). There is a great need for comprehensive community-based programs that focus on promoting social inclusion and raising awareness about the challenges and stigmas widows face. Most literature on support for widows refers to the WHR's advocacy for Nepalese widows from a social justice perspective. WHR's primary goals have been to educate the community and improve Nepalese widows' legal and social-economic rights and medical health care access. WHR's initiatives have been focused on diversity, equity, and inclusion for Nepalese widows, identifying widows' resiliency, building on their self-esteem and self-confidence, and making sure widows are included in public health policy initiatives (4).

WHR reports several programs that contribute to the overall development of the single woman. These include monthly sharing meetings and access to opportunities such as *SWEG* (Single Women Entrepreneurs' Group) for vocational training; *Aadhar Cooperative* for microcredit (Savings & Credit); *Opportunity Fund* for educational scholarships for women and their children, and *Raahat* for rehabilitation of single women facing domestic violence (4). WHR's extensive initiatives to empower Nepalese widows are the most pivotal contributions in the Nepalese widow's movement history, but much more is needed. The WHR emphasizes the need for collaboration between private and public sectors of society, as well as support from local and national government. With regard to the younger generation, the WHR states, “There is hope that the younger generation will internalize the problems faced by the widows and that through their awareness, the harmful cultural practices and traditions adversely affecting widows will disappear” (4).

## Conclusions

This study addressed the high levels of anxiety, depression, and stress related symptoms among Nepalese widows. Additionally, this study examined the relationship of certain demographic variables with these mental health symptoms. Widows in Nepal were shown to experience high symptoms levels relative to established cut-points and to existing normative data for female nonpsychiatric and psychiatrics samples and a Nepalese older women's sample. The age of the widows had an inverse relationship with their levels of stress related symptoms. Research on this topic is still developing in Nepal. There are factors that perpetuate the development of mental health symptoms; these include stigma of mental health treatment, limited number of treatment facilities for individuals with lower incomes, gender roles and patriarchy, and widowed status. Economic status, gender and widowed status continue to oppress Nepalese widows, as evidenced by lack of necessary access to medical, financial, and mental health services. Government initiatives and public policies are needed to support and strengthen these minoritized communities. Since there is a lack of emphasis on education and obtaining employment for women in South Asian countries, many widows are not aware of the legal rights and support they can receive. Thus, there is a need for development of more non-governmental organizations such as the Women for Human Rights (4) that take a social justice advocacy approach for past and current widows and for future generations to come.

## Data Availability

The raw data supporting the conclusions of this article will be made available by the authors, without undue reservation.
